# Hospital Meetings

**Published:** 1901-05-25

**Authors:** 


### HOSPITAL MEETINGS.

### VICTORIA HOSPITAL FOR CHILDREN.

Mr. F. W. Hunt presided at the thirty-fourth Annual Court of Governors of the Victoria Hospital for Children, Chelsea and Broadstairs, on the 15th inst.

In their report the committee of management expressed their gratification that during the past year the hospital had not had to be closed, the work done consequently showing considerable increase when compared with the previous twelve months. Special precautions had been taken to guard against the spread of infectious diseases, for advice and assistance in regard to which thanks were due to Dr. Louis Parkes. The in-patients numbered 560 and the out-patient attendances 48,390, 16,967 being new cases; 453 children were sent to the Broadstairs Convalescent Home. The debt to the bankers had had to be increased from £500 to £1,250. The ordinary income for the year was £5,280, an increase of £736. The extraordinary income, however, was only £30 as compared with £1,552 in 1899. There was a considerable falling off in the profit from private nursing, and the committee expressed the hope that friends would help them by employing and recommending the trained nurses supplied by the institution. The ordinary expenditure was £5,349—a slight increase on that of last year—and the extraordinary expenditure £700 (to meet the deficit on the Convalescent Home at Broadstairs). The income of the Convalescent Home, including £203 from patients' payments, was £697 and the expenditure £1,397. A very large proportion of the children sent there returned either cured or greatly benefited. In many cases they remained two or three months, and in one instance for six months. The committee greatly regretted not having been able to commence work on the much needed new building owing to want of funds. The amount at their disposal had now reached £6,221, but they did not feel justified in beginning work until a much larger proportion of the requisite £25,000 was forthcoming. The Prince of Wales's Fund had called attention to the fact that the building had not been commenced, the £500 which they granted in January, 1900, having been given on the understanding that steps were to be taken during the year. So that a special effort should be made in this direction it had been arranged to hold a Festival Dinner in the autumn, at which Lord Roberts had consented to preside. Regret was expressed at the loss to the institution by the death of Sir Arthur Sullivan. Mr. Herbert Sullivan had, however, promised to continue to support the "Sullivan Cot" which was founded in 1877, and maintained by yearly subscription. During the year Dr. W. J. Fenton was appointed physician to out-patients in place of Dr. Arthur C. Latham. Mr. J. F. Ridley was elected a member of the committee of management. The Hon. R. A. Lubbock had among other gifts presented a new antiseptic operating table and as an acknowledgment had been elected a vice-president. Mr. A. Cameron Skinner resigned his position as secretary owing to ill-health, and Mr. H. G. Evered (formerly secretary to the Invalid Children's Aid Association) was on July 4th elected to the post.

The report was adopted, the secretary reading the remarks on it which had been prepared by Mr. Martin Smith, he being prevented by illness from being present.

The Lord Bishop of London was elected a vice-patron of the hospital in succession to the late Bishop Creighton; the retiring members of the committee were re-elected; and Mr. E. A. Dowse was elected hon. solicitor in the place of his father who had resigned owing to ill-health. A resolution of thanks to Mr. H. A. Dowse for his valuable services as hon. solicitor, expressing the hope that he would soon be restored to health was also passed; a vote of thanks to the chairman concluding the proceedings.

### NORTH-EASTERN HOSPITAL FOR CHILDREN.

The 33rd annual meeting of the North-Eastern Hospital for Children was held on the 17th inst., the Rev. W. G. Morcom presiding.

The report presented stated that the number of in-patients during the past year was 778, and of out-patients 18,264, with 62,931 attendances. These numbers compared with 748 in-patients and 16,665 out-patients, with 59,203 attendances in 1899, when the hospital was closed for several weeks. The receipts for the year, including £81 for the Convalescent Fund, amounted to £6,365, and the expenditure, including £60 on account of the Convalescent Fund, was £6,257. The deficit of £28 carried forward from the previous year had thus been converted into a credit balance of £80. In addition to their annual subscription of £500, the Prince of Wales's Fund gave a further special donation of £500 to the Building Fund, and one of £50 to the General Fund, with a view to the abolition of the charge of 6d. per week made for in-patients' washing. The legacies during the year amounted to £160, of which £100 was placed to the building fund. For this fund an appeal was made in November which resulted in the receipt of £3,047, bringing the amount of the fund up to £9,848 on December 31st. A festival dinner in aid of the fund had been arranged for the 21st inst. in the Haberdashers' Hall, at which Sir Marcus Samuel had consented to preside.

During the year Mr. Henry Allhusen, M.P., Sir John Knill, and Mr. Abram Lyle had been elected to fill vacancies on the committee. The death was recorded of Dr. F. Charlewood Turner, physician to the Hospital from 1877 to 1893, and after that a member of the consulting staff. Mr. E. W. Clapham had been appointed resident medical officer in succession to Mr. C. G. Burton. The system of inquiry into the circumstances of out-patients commenced in 1898 had continued to work satisfactorily, many unsuitable persons having been prevented from resorting to the hospital. Extended inquiries were made in 107 cases, and of these 50 were considered to be unsuitable.

The Chairman moved the adoption of the report and emphasised the need for the enlargement of the hospital. There were something like a quarter of a million children forming their constituency, many of the families being crowded into a single apartment, and in very many cases inability to get into the hospital was practically equivalent to a death sentence. The report was adopted.

Mr. Walter Johnson moved the election of the vice-presidents, treasurer, and committee for the year, and in doing so referred in detail to the scheme of enlargement which he said would entail an expenditure of £40,000. After having secured the freehold of the necessary land adjoining they had some £10,000 in hand towards the cost of building. When completed the enlargement would necessitate the expenditure of some £3,000 a year beyond the £6,000 at present being spent. The committee of management were full of hope for the future. They were receiving the support of many local societies which was particularly valuable as being the result of the efforts of men who lived in the neighbourhood, who knew the needs of the locality and how they were being met by the hospital. From the Hackney Society in aid of the Children's Hospital they had received during the year £360, from the Central Hackney Musical and Benevolent Society £326, and from the North-Eastern Hospital Aid Society £167.

The Chairman mentioned that no decision had yet been come to with regard to the question of a president for the institution, but the matter was receiving careful attention.

Votes of thanks concluded the proceedings.

### ALEXANDRA HOSPITAL.

The thirty-fourth annual meeting of the Alexandra Hospital for Children with Hip Disease was held on the 16th instant, Mr. W. H. Whitfield, the treasurer and chairman of the committee, presiding. The report and accounts presented showed that the income for the past year (including £814 special subscriptions, and interest on such, for the maintenance of cots) was £3,872 and the expenditure £3,524, there being thus a credit balance of £348. Annual subscriptions showed a slight increase, totalling £1,275 as against £1,237 in 1899. Donations, however, were less, being £1,114 as compared with £1,260. The surplus of income over expenditure was to some extent accounted for by the fact that all the beds in the new hospital had not yet been fully occupied. There were two wards still unopened, but all liabilities in connection with the building and furnishing had been discharged. It was hoped that the remaining 20 beds would be at the disposal of patients during this year. The report was adopted; the retiring members of the committee were re-elected, and the election of Sir William Graham and Mr. P. J. Crichton-Stuart confirmed. The trustees, medical officers, auditors, and treasurer were re-elected, and votes of thanks brought the meeting to a close.

### CHELSEA HOSPITAL FOR WOMEN.

The annual meeting of the above hospital took place on Tuesday, the 21st inst. The Lord Glenesk presided. He alluded to the death of H.M. Queen Victoria, which he thought people hardly yet realised. The course of the hospital had been one of continuous good. At one time it seemed as if its resources would be overtaxed, but through the kindness of a former benefactor this was averted: he had given a third donation of £1,000. The Prince of Wales' Hospital Fund had given £350, and the authorities had made only one suggestion with regard to improvements: they wished that the wards should be re-floored, and towards the expense they had given £250. The work would be carried out during the summer, and would be according to the most recent sanitary ideals. The floors would be of wood, very highly polished. The cost would be about £750. This was not the lowest estimate, but the work would be completed in a shorter time than if they had accepted the lowest estimate. The City companies had been very generous, and altogether close upon £500 had been promised; for the rest, the generosity of friends must be relied upon. It was a necessary outlay, and would make the hospital almost, if not quite, perfect. It had been decided to close the hospital, on the recommendation of the medical staff, for a summer holiday, so that all the painting, cleaning, and flooring could be accomplished at one time without disturbing the patients. Although some of the annual subscriptions had fallen off, new friends had come forward; he hoped their example would be followed by newer ones still. The death-rate in the hospital had a continuous tendency to decrease; it was a remarkable fact that Dr. Duncan had performed 137 consecutive cases of major operations with only one death; on the total number of major operations the deaths had been 3 per cent., and this, even in the light of modern science, was very low. The Ladies' Committee continued their kind services, which were much appreciated by the patients. Dr. Ridgeway (now Bishop of Kensington) had been succeeded as Hon. Chaplain by the Rev. W. S. Swayne. The Convalescent Home had had a prosperous year; 166 patients had been sent to St. Leonards to complete their recovery by change of air and scene, good food, and happy surroundings. It had been decided not to admit cases of consumption, since that disease was now looked upon as infectious.

Mr. H. E. Wright, treasurer, seconded the adoption of the report, which was carried unanimously.

Dr. Duncan proposed the re-election of the Council, and Lady Fox Young seconded this.

Mr. J. S. Wood, in moving a vote of thanks to the medical staff, said that the low death rate was a very remarkable thing; it meant that whereas some few years ago 95 patients died, 97 now live; it was an extraordinary proof of the advance of surgery. Speaking of the relations between the committee and the medical staff, he said those at present engaged in controversy on this point might well refer to Chelsea Hospital for Women. The medical staff had a separate committee, with their own minutes and their own secretary; they sent recommendations to the council, and the council in turn referred to them. He would like to recommend this plan as one which worked harmoniously. Two ladies were on the hon. medical staff, as anæsthetist and assistant anæsthetist—another arrangement which worked very satisfactorily.

